# B cell-derived circulating granzyme B is a feature of acute infectious mononucleosis

**DOI:** 10.1038/cti.2015.10

**Published:** 2015-06-26

**Authors:** Magdalena Hagn, Archana Panikkar, Corey Smith, Henry H Balfour Jr, Rajiv Khanna, Ilia Voskoboinik, Joseph A Trapani

**Affiliations:** 1 Cancer Immunology Program, Cancer Cell Death Laboratory, Peter MacCallum Cancer Centre, East Melbourne, Victoria, Australia; 2 Sir Peter MacCallum Department of Oncology, The University of Melbourne, Parkvillie, Victoria, Australia; 3 Centre for Immunotherapy and Vaccine Development, Queensland Institute of Medical Research, Brisbane, Queensland, Australia; 4 Department of Laboratory Medicine and Pathology, Paediatrics and Microbiology, University of Minnesota Medical School, Minneapolis, Minnesota, USA; 5 Victorian Comprehensive Cancer Centre, Melbourne, Victoria, Australia

## Abstract

Granzyme B (GzmB) is a serine protease best known for inducing target cell apoptosis when released by cytotoxic T lymphocytes (CTLs) or natural killer cells with pore-forming perforin. As a result, GzmB detected in the serum of virus-infected individuals has typically been attributed to these sources. Here, we show that patients with recently diagnosed infectious mononucleosis caused by Epstein-Barr virus (EBV) have high circulating levels of GzmB that may be derived from infected B cells early in course of disease. We recently reported that human B cells from healthy donors secrete active GzmB when stimulated *in vitro* through B-cell receptor (BCR) ligation and interleukin (IL)-21. We found that infecting B cells with EBV greatly amplified GzmB secretion in response to the same stimuli, but the expression was terminated once the infection had become latent. Our results represent a rare instance of GzmB expression by non-CTL/natural killer cells in the context of infection with a human pathogen.

Epstein-Barr virus (EBV) is a highly contagious, orally-transmitted pathogen that infects most of the population during childhood or adolescence. Childhood infections are typically mild and self-limiting but adolescents and adults may develop infectious mononucleosis (IM), a syndrome marked by pharyngitis, severe local lymphadenopathy, splenomegaly and prolonged lethargy.^1^ Upon reaching the circulation, EBV readily infects resting B cells, and ‘atypical' mononuclear cells are frequently noted on blood smears. Following clearance by cytotoxic T lymphocytes (CTLs), the virus remains ‘dormant' in memory B cells for life, in immune-competent hosts. If EBV reactivates, then anti-EBV CTL recognizes and destroy infected B cells through the perforin/granzyme B (GzmB)-dependent apoptotic pathway.^2, 3^ Although CTL and natural killer cells are considered as the major cellular source of GzmB, human B cells can also release large amounts of active GzmB when stimulated *in vitro* with interleukin-21 (IL-21) and B-cell receptor (BCR) cross-linking.^4, 5^ In this study, we showed that GzmB is readily detectable in the serum of patients with acute EBV-related IM and is likely to be transiently secreted by infected B cells. This is the first report of a pathophysiological context for GzmB expression and release by B lymphocytes.

## Results and discussion

**Figure 1:**
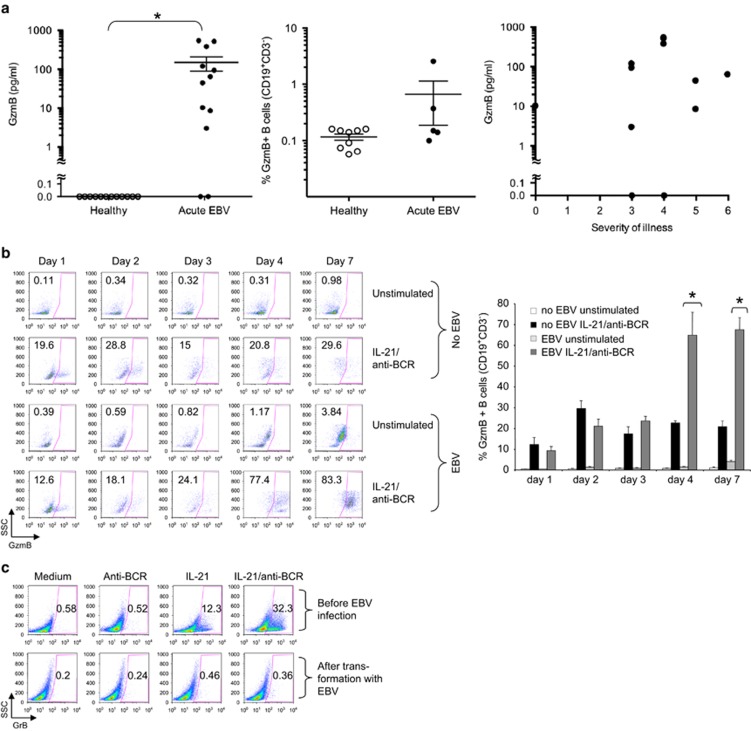
Patients with freshly diagnosed IM show elevated serum levels of granzyme B, likely due to large secretion from B cells during acute, but not chronic infection with EBV. After obtaining informed consent, peripheral blood and serum was taken from healthy donors or patients with recently diagnosed IM, as determined by detection of anti-VCA-IgM, or age-matched EBV+ healthy controls (*n*=12 each group). (**a**) Left panel shows results of serum levels of GzmB in each donor, as determined by cytometric bead array (BD Biosciences, San Jose, CA, USA). Horizontal lines represent mean levels in each group±s.e.m. *Indicates two-sided *P-*value <0.0001 for the difference between patients and healthy individuals by Mann–Whitney *U* test. Middle panel shows percentages of GzmB^+^ B cells in patients and healthy controls as determined by intracellular flow cytometry (mean±s.e.m.). Cells were harvested following 4 h incubation with Brefeldin A (1 μg ml^−1^), fixed, permeabilized, and GzmB expression was assessed using a PE-labelled GzmB antibody (1:400, Sanquin, Amsterdam, The Netherlands). Cell-surface markers were analysed using anti-CD3 and anti-CD19 (BD Biosciences). Right panel shows the severity of illness index in each patient compared with GzmB levels. (**b**, **c**) PBMC from healthy donors were isolated and either infected with the B95.8 strain of EBV or left untreated. After infection, cells were plated in 48-well plates at 1 × 10^6^ cells per ml and stimulated for 16 h with or without human recombinant IL-21 (50 ng ml^−1^, Invitrogen, Carlsbad, CA, USA) and 6.5 μg ml^−1^ anti-BCR (affinity purified rabbit F(ab')_2_ against human IgA+IgG+IgM (H+L); Jackson ImmunoResearch Laboratories, West Grove, PA, USA) on the time points indicated. Next, cells were harvested on indicated time points after infection, and GzmB expression was assessed by intracellular flow cytometry. (**b**) Left panel: dot plots show percentages of GzmB^+^ CD19^+^ CD3^−^ B cells. Right panel: bar graphs depict summarized results of five independent experiments. Error bars show±s.e.m., *Indicates *P*<0.01 by Student's *t*-test. (**c**) Dot plots show GzmB^+^ B cells from freshly isolated B cells from a healthy volunteer (purity >99%, upper panel, negative selection, Miltenyi Biotech, Bergisch Gladbach, Germany) or after full *in vitro* transformation with the B95.8 strain of EBV (4–5 weeks after infection). B cells were stimulated for 16 h with either IL-21 or anti-BCR or both. Successful EBV transformation was determined by observation of distinct morphological feature changes by microscopy and by expression of EBNA-2 protein in B cells by western immunoblot (not shown).

We measured plasma GzmB levels in 12 patients with active EBV-related IM and age-matched EBV-seropositive healthy individuals. GzmB was undetectable in control subjects but measurable in 10/12 patients (mean 150.2 pg ml^−^^1^±60.4 s.e.m., range 3–548 pg ml^−1^; *P*<0.0001, Figure 1a, left panel). The highest levels were found in patients with intermediate symptomatology (as previously defined^6^), whereas patients at either end of the disease severity spectrum had lower levels, including 3/3 with severe symptomatology that had low GzmB levels (Figure 1a, right panel). A study that includes a larger number of patients will be necessary to confirm this finding. However, our current data suggest that circulating GzmB is unlikely to be a ‘non-specific' marker of systemic inflammation associated with IM.

GzmB has been previously detected in the serum of 14 patients at various stages of EBV infection; however, these levels were not significantly different from healthy controls.^7^ In that study, circulating GzmB was hypothesized to derive from EBV-specific CD8^+^ CTL responses. However, as human B lymphocytes can express GzmB in an IL-21-dependent manner early during viral infection, following routine immunization^4^ or in autoimmune diseases such as systemic lupus erythematosus,^8^ we examined B cells as a possible source of GzmB in IM patients. To test this, we infected B cells of healthy adult volunteers with EBV *in vitro*, and tested them for GzmB expression in response to IL-21/anti-BCR. These cells expressed far higher levels of GzmB than mock-infected controls stimulated in the same way. By intracellular FACS staining, the number of GzmB-expressing B cells was similar over the first 2 days, but increased dramatically in EBV-infected cells after 4 days (Figure 1b; *N*=5, *P* <0.01). The EBV-infected B cells failed to produce GzmB if IL-21/anti-BCR addition was delayed until day 10; chronically EBV-infected lymphoblastoid cell lines produced no GzmB (Figure 1c). These data indicated that GzmB expression can only be induced in B cells at specific (early) stages of EBV infection and is a transient feature of acute disease. This may also explain the considerable variability of GzmB levels in our patient cohort, with some patients having been tested soon after infection, and others days or weeks later.

To our knowledge, this is one of the first reports of elevated circulating GzmB in an acute viral illness, where the granzyme originates from infected cells, in this case B lymphocytes, not CTLs. Similarly, a recent study showed a novel regulatory B cell in patients with HIV characterized by potent GrB-dependent T cell-suppressive activity.^9^ This effect was associated with the GrB-dependent cleavage of the T cell receptor zeta chain, which has been shown to be a GrB substrate.^10^ In the pathogenesis of IM, however, the specific role of circulating GzmB is unknown. In the absence of perforin, it is highly unlikely to have a pro-apoptotic effect on other cells. Also, we found no evidence *in vitro* that GzmB induces B-cell suicide in response to EBV infection.^4^ Rather, the GzmB was rapidly secreted,^4^ and the infected cells continued to proliferate, while also expressing high levels of the GzmB serpin inhibitor, Serpin B9 (Supplementary Figure 1). This may also explain the low levels of constitutive GzmB in peripheral B cells of our patients, despite being increased compared with healthy controls (Figure 1a, middle panel).

As well as being pro-apoptotic, GzmB has a range of extracellular functions including matrix degradation, cleavage of cell-surface receptors, and modulation of inflammatory pathways (reviewed in Hiebert and Granville^11^). Furthermore, GzmB has been reported to induce cell detachment in the absence of perforin, via degradation of extracellular matrix proteins.^12, 13^ Theses studies also proposed that GzmB might attract a variety of immune cell types, playing a role in local tissue inflammation.^14^ Our study provides a possible source for extracellular GzmB in IM. Typically, cytotoxic lymphocytes release GzmB into the immune synapse with peforin, which enables it to access the target cell cytosol. In contrast, GzmB release from B cells is not accompanied by perforin,^4^ and the fact that it remains active in plasma^13^ makes an extracellular function for GzmB more feasible. GzmB can cleave basement membrane proteins and promote migration of CTLs into inflamed tissue, and thus, in acute EBV infection, may facilitate the T-cell response to the virus.^15^ Conversely, EBV might utilize this ‘pulse' of B cell-derived GzmB induced by recognition of the virus through the B cell's BCR, and IL-21 deriving from follicular helper T cells in nearby germinal centres to modify bystander cells, facilitating further virus uptake and replication.^5^

## Methods

### Human subjects and *in vitro* EBV infections

This project had ethics approval of the Peter MacCallum Cancer Centre (No. 12/73, age range 18–40 years), the QIMR Berghofer Medical Research and Uniting Care Health Human Research Ethics Committees, and by the Research Subjects Protection Program of the University of Minnesota. After obtaining informed consent, peripheral blood was taken from healthy donors or patients with recently diagnosed IM, as determined by detection of anti-VCA-IgM, or age-matched EBV+ healthy controls. Serum was collected and peripheral blood mononuclear cells were isolated by Ficoll density gradient. Primary B cells were cultured in RPMI-1640 supplemented with 10% FBS in 48-well plates at 1 × 10^6^ cells per well per ml, if not stated otherwise. For infection with EBV, B cells were incubated for 2 h at 37 °C with B95.8 virus (kindly provided by Joanne Davis, Royal Melbourne Hospital, Melbourne, Australia), washed and plated as indicated in the figure legends. Human recombinant IL-21 (Invitrogen) was added at 50 ng ml^−1^. Polyclonal B-cell stimulation was achieved with affinity purified rabbit F(ab')_2_ against human IgA+IgG+IgM (H+L) at 6.5 μg ml^−1^ (anti-BCR; Jackson ImmunoResearch Laboratories, West Grove, PA, USA).

### Cytometric bead array

The CBA Flex Systems Kit (BD Biosciences, San Jose, CA) was used to measure GzmB according to the manufacturer's instructions.

### Flow cytometry

After culture, B cells were harvested, fixed and permeabilized as described previously.^4^ For intracellular GrB staining, a PE-labeled antibody (1:400, clone GB11; Sanquin) was used. Cell-surface markers were analysed in some experiments using CD3-PacificBlue and CD19 Pe-Cy7 (BD Biosciences). Data were acquired on a FACSCanto or LSR device (BD Biosciences) and analysed using FlowJo software (version 8.8.7; Tree Star, Stanford, CA, USA).
