# Supplementary material for: B cell-derived circulating granzyme B is a feature of acute infectious mononucleosis
Source: Clin Transl Immunology. 2015 Jun 26;4(6):e38–. doi: 10.1038/cti.2015.10 (PMC4491623; doi:10.1038/cti.2015.10)
Supplement: Supplementary Information [file cti201510x1.doc]

**Supplementary Material and Methods**

**Western Blot**

B cells (purity >99%) were isolated by negative selection (Miltenyi, Bergisch Gladbach, Germany) and stimulated for 16 hrs with IL-21 (50 ng/ml) and anti-BCR (6.5µg/ml), or a combination of both. After stimulation, B cell lysates were prepared, and protein content was measured with a Bradford assay (Thermo Scientific, Waltham, MA, USA USA). Proteins were separated on a 4-12% SDS-PAGE gradient gel (NuPage® Bis-Tris Mini gels, Invitrogen) and transferred onto 0.45-mm PVDF membranes. After washing and blocking, membranes were incubated over night at 4° C with the following antibodies: mouse anti-human GrB (1:1000, clone 2C5, in house), mouse anti-human SERPINB9 (1:1000, clone 7D8, in house), and mouse anti-human β-actin (1:2000, clone AC-74, Sigma Aldrich). Membranes were washed, incubated for 1 hour at room temperature with rabbit anti-mouse Ig/HRP (1:1000, Dako, Glostrup, Denmark), and developed.

**Flow cytometry**

Highly purified B cells (>99%) were stimulated for 16 hrs with IL-21 (50 ng/ml) and anti-BCR (6.5µg/ml), or a combination of both. Intracellular flow cytometry was performed using antibodies for GzmB (1:400, clone GB11; Sanquin, Amsterdam, Netherlands) and SERPINB9 (1:50, clone 7D8, AbD Serotec, Oxford, UK). Data were acquired on a FACSCanto or LSR device (BD Biosciences) and analyzed using FlowJo software (version 8.8.7; Tree Star, Stanford, CA).

**Real-time (RT)-PCR**

After culture, RNA of highly purified B cells (>99%) was isolated using an RNeasy mini kit (Qiagen, Doncaster, Australia) according to the manufacturer’s protocol. RNA was transcribed with M-MLV Reverse Transcriptase (RNase H Minus, Point Mutant) and oligonucleotide primer (both from Promega, Madison, WI). RT quantitative PCR was performed in a Corbett Rotor Gene 6000 cycler (Corbett Life Sciences, Concorde, Australia), using SYTO9 green fluorescent nucleic acid stain (Invitrogen). The following primers were used (all Sigma):

Table I: Primer sequences for real-time RT PCR

| **Gene** | **Sequence (5’ to 3’)** |
| --- | --- |
| GzmB | F: ccatccagcctataatccta |
|  | R: cctgcactgtcatcttcacct |
| RPL-32 | F: gcgtaactggcggaaaccca |
|  | R: ttgtgagcgatctcggcaca |
| SERPINB9 | F: GGCATTTGGGAATTGTTGATG |
|  | R: ACAGGTCTCTCTCCGCTGACA |

F: forward primer; R: reverse primer

Samples were run in duplicate and mRNA expression of the genes of interest normalized to the housekeeping gene RPL-32 using Rotor Gene 6000 Software (version 1.7) and Microsoft Excel 2011. Relative expression levels compared to control were calculated as 2(^-ΔΔCt^).
