# Supplementary material for: B cell-derived circulating granzyme B is a feature of acute infectious mononucleosis
Source: Clin Transl Immunology. 2015 Jun 26;4(6):e38–. doi: 10.1038/cti.2015.10 (PMC4491623; doi:10.1038/cti.2015.10)
Supplement: Supplementary Figure Legends [file cti201510x2.doc]

**Supplementary Figure 1. IL-21/anti-BCR induce *de novo* GzmB in B cells whereas Serpin B9 is constitutively expressed.**

B cells (purity >99%) were isolated by negative selection (Miltenyi, Bergisch Gladbach, Germany) and stimulated for 16 hrs with IL-21 (50 ng/ml) and anti-BCR (6.5µg/ml), or a combination of both. Non B cells were stimulated for 16 hrs with 1µg/ml PHA. (A) Cell lysates were prepared and GzmB (1:1000, clone 2C5, in house) and SERPINB9 (1:1000, clone 7D8, in house) analysed by Western Blot. (B) After culture, cells were incubated for 4 hrs with Brefeldin A and subsequently stained with antibodies against GrzmB (1:400, Sanquin, Amsterdam, Netherlands) and SERPINB9 (1:50, clone 7D8, AbD Serotec, Oxford, UK). Dot plots depict percentages of intracellular GrzmB and SERPINB9 in B cells as examined by flow cytometry (C). Total RNA was prepared (RNeasy mini kit, Qiagen, Doncaster, Australia) and real-time-PCR performed on expression of GrzmB and SERPINB9 in a Corbett Rotor Gene 6000 cycler (Corbett Life Sciences, Concorde, Australia), using SYTO9 green fluorescent nucleic acid stain (Invitrogen). Bar graphs represent relative quantity compared to unstimulated B cells as determined by the relative CT method. All data are representative of at least three independent experiments. Error bars indicate +SEM.
