# Supplementary material for: B cell-derived circulating granzyme B is a feature of acute infectious mononucleosis
Source: Clin Transl Immunology. 2015 Jun 26;4(6):e38–. doi: 10.1038/cti.2015.10 (PMC4491623; doi:10.1038/cti.2015.10)
Supplement: Supplementary Figure 1 [file cti201510x3.ppt]

## Slide 1
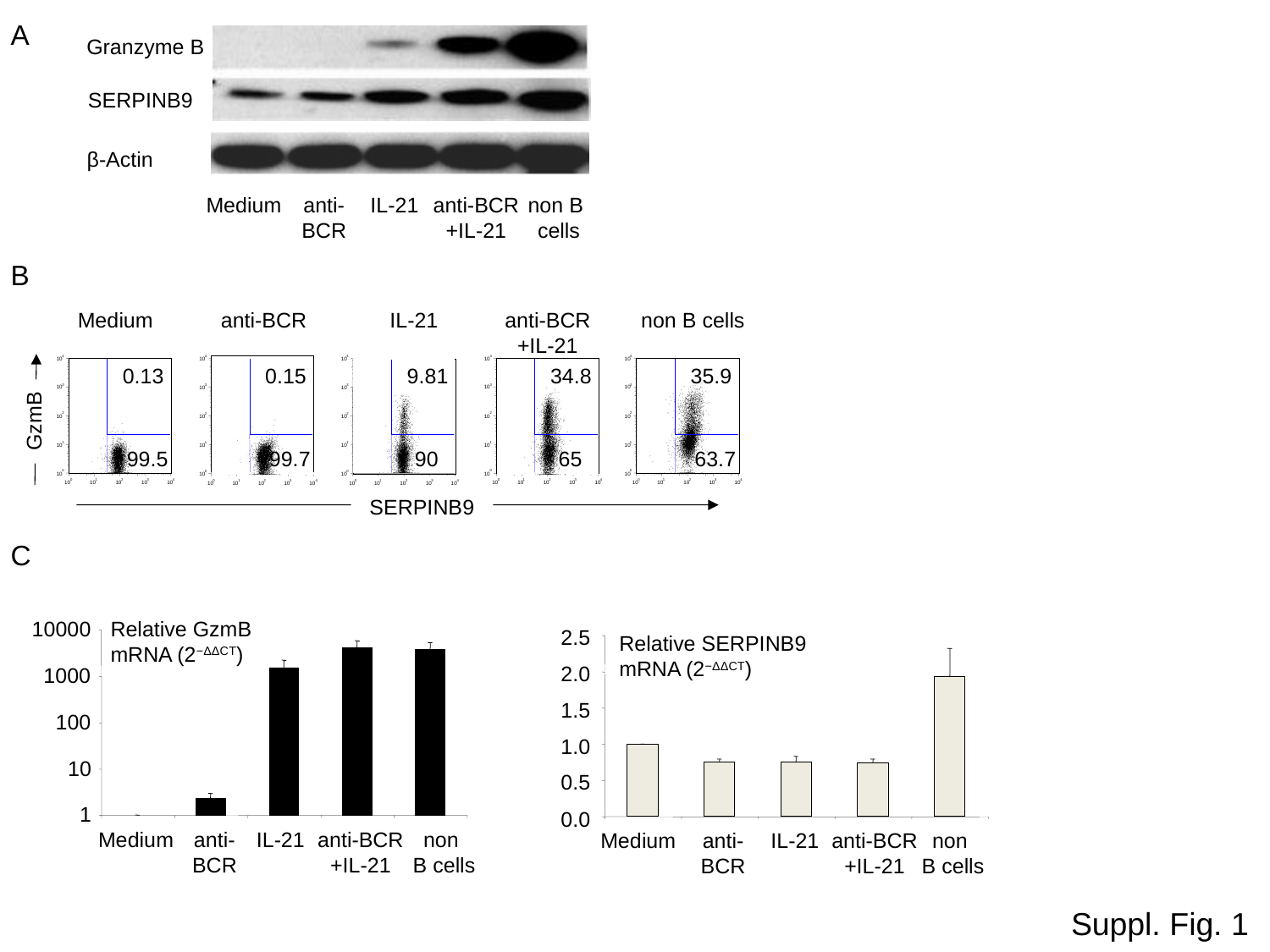

A
Granzyme B
SERPINB9
β-Actin
Medium
anti-
BCR
IL-21
anti-BCR
+IL-21
non B
 cells
B
Medium
anti-BCR
IL-21
anti-BCR
+IL-21
non B cells
4
4
4
4
4
10
10
10
10
10
0.13
0.15
9.81
34.8
35.9
3
3
3
3
3
10
10
10
10
10
GzmB
2
2
2
2
2
10
10
10
10
10
1
1
1
1
1
10
10
10
10
10
99.5
99.7
90
65
63.7
0
0
0
0
0
10
10
10
10
10
0
1
2
3
4
0
1
2
3
4
0
1
2
3
4
0
1
2
3
4
0
1
2
3
4
10
10
10
10
10
10
10
10
10
10
10
10
10
10
10
10
10
10
10
10
10
10
10
10
10
 SERPINB9
C
Relative GzmB mRNA (2−ΔΔCT)
10000
1000
100
10
1
Medium
anti-
BCR
IL-21
anti-BCR
+IL-21
non
B cells
2.5
Relative SERPINB9 mRNA (2−ΔΔCT)
2.0
1.5
1.0
0.5
0.0
Medium
anti-
BCR
IL-21
anti-BCR
+IL-21
non
B cells
Suppl. Fig. 1
